# Androgen Receptor Activity Is Affected by Both Nuclear Matrix Localization and the Phosphorylation Status of the Heterogeneous Nuclear Ribonucleoprotein K in Anti-Androgen-Treated LNCaP Cells

**DOI:** 10.1371/journal.pone.0079212

**Published:** 2013-11-13

**Authors:** Paola Barboro, Luana Borzì, Erica Repaci, Nicoletta Ferrari, Cecilia Balbi

**Affiliations:** 1 Translational Urologic Research Unit, IRCCS Azienda Ospedaliera Universitaria San Martino IST-Instituto Nazionale per la Ricerca sul Cancro, Genova, Italy; 2 Molecular Oncology and Angiogenesis Unit, IRCCS Azienda Ospedaliera Universitaria San Martino IST-Istituto Nazionale per la Ricerca sul Cancro, Genova, Italy; Clermont Université, France

## Abstract

The androgen receptor (AR) plays a central role in the development and progression of prostate cancer (PCa) and anti-androgen therapy is a standard treatment. Unfortunately, after a few years, the majority of patients progress, developing androgen-independent PCa. AR-driven gene transcription recruits a large number of co-activator/co-repressor complexes; among these, the heterogeneous nuclear ribonucleoprotein K (hnRNP K) directly interacts with and regulates the AR translational apparatus. Here we examined AR and hnRNP K expression in response to the treatment of LNCaP cells with anti-androgen cyproterone acetate (CPA) or bicalutamide (BIC). AR and hnRNP K modulation and compartmentalization were studied by Western blot and confocal microscopy. Phosphate-affinity gel electrophoresis was employed to examine how anti-androgens modified hnRNP K phosphorylation. 10^−6 ^M CPA significantly stimulated LNCaP proliferation, whereas for 10^−4 ^M CPA or 10^−5 ^M BIC an antagonistic effect was observed. After anti-androgen treatment, AR expression was remarkably down-regulated within both the cytoplasm and the nucleus; however, when CPA had an agonist activity, the AR associated with the nuclear matrix (NM) increased approximately 2.5 times. This increase was synchronous with a higher PSA expression, indicating that the NM-associated AR represents the active complex. After BIC treatment, hnRNP K expression was significantly lower in the NM, the protein was hypophosphorylated and the co-localization of AR and hnRNP K decreased. In contrast, CPA as an agonist caused hnRNP K hyperphosphorylation and an increase in the co-localization of two proteins. These findings demonstrate that, in vitro, there is a strong relationship between NM-associated AR and both cell viability and PSA levels, indicating that AR transcriptional activity is critically dependent on its subnuclear localization. Moreover, the agonistic/antagonistic activity of anti-androgens is associated with modifications in hnRNP K phosphorylation, indicating an involvement of this protein in the AR transcriptional activity and likely in the onset of the androgen-independent phenotype.

## Introduction

Prostate cancer (PCa) is currently a leading cause of morbidity in the western male population [1], and it is known that the androgen receptor (AR) plays a central role in the development and progression of this tumor [2]. Because PCa growth is initially androgen dependent, anti-androgen therapy, in combination with surgical or medical castration, is the standard treatment. Two structurally distinct drug types are in common use: steroidal and non-steroidal [3]. In both cases, androgen deprivation initially leads to tumor remission; however, after a few years of treatment, the majority of patients progress and develop androgen-independent PCa, a lethal form of the disease, due to a lack of effective therapies. Little is known regarding how anti-androgens exert their effects, and several pathways have been proposed to explain androgen independence; however, the mechanisms responsible for its emergence remain unclear [4].

AR-mediated gene transcription involves the recruitment of a large number of co-activator/co-repressor complexes, and it has recently been demonstrated that the heterogeneous nuclear ribonucleoprotein K (hnRNP K) directly interacts with and regulates the AR translational apparatus [5]. In human and murine PCa cells, hnRNP K and AR colocalize in the nucleoplasm in a complex that is highly proximal to DNA, and treatment with bicalutamide (BIC) and/or 4-hydroxy-tamoxifen results in anomalous hnRNP K phosphorylation and in a consequent modulation of the complex [6]. Utilizing a proteomic approach, we demonstrated that the expression of a hyperphosphorylated hnRNP K isoform present in the nuclear matrix (NM) is strongly related to both the PCa diagnosis and the clinical outcome of patients after radical prostatectomy [7], [8]. Moreover, the AKT/hnRNP K/AR/β-catenin pathway is critical for the acquisition of the neuroendocrine phenotype that is associated with a more aggressive PCa and correlates with poor prognosis [9]. These results suggest that hnRNP K and its interaction with AR play a role in PCa development and progression.

It is known that the unbound AR resides predominantly in the cytoplasm in a complex containing heat-shock proteins; the presence of androgen initiates a cascade of events that leads to receptor dimerization and translocation into the nucleus. Interaction of the AR with anti-androgens has been intensely investigated; however, the precise molecular mechanisms of their action remain unclear. Little is known regarding the way by which these drugs influence AR subnuclear localization and the dynamics of coactivator recruitment. Therefore, in this study, we examined the distribution of AR and its regulator hnRNP K in the cytoplasm, nucleus and NM in response to treatment of androgen-sensitive LNCaP cells with the steroidal anti-androgen cyproterone acetate (CPA) and the non-steroidal BIC. The elucidation of the mechanisms underlying AR suppression may provide a basis to understand the pathways involved in PCa progression and in the development of androgen escape.

## Materials and Methods

### Cell Culture

LNCaP cells were obtained from ATCC (CRL-1740; Rockville, MD, USA) and maintained in RPMI-1640 medium without phenol red (Celbio, Milan, Italy) containing heat-inactivated 10% fetal bovine serum (charcoal stripped), 100 U/ml penicillin G, 34 µM streptomycin, 2 mM glutamine, 10 mM HEPES, 1 mM sodium pyruvate and 25 mM glucose (Celbio). The passage numbers at which these cells were placed on maintenance medium ranged from 24 to 34. Control cells were cultured in monolayer in the presence of 0.1 nM 5-α-dihydrosterone (DHT; SIGMA, St. Louis, MO, USA) for 72 h at 37°C in 5% CO_2_. Subsequently, the medium was changed, and the cells were incubated for 48 or 72 h with BIC (SIGMA) or CPA (SIGMA) in the presence of 0.1 nM DHT. To elucidate the phosphorylation status of hnRNP K, LNCaP cells were also treated with 200 nM Wortmannin (SIGMA) for 5 h. For each treatment, viable cell numbers were determined using an MTT assay according to the methods recommended by the manufacturer (SIGMA). Absorbance was recorded at 595 nm, and the data are presented as the percentages of the absorbance of BIC- or CPA-treated cells with respect to control cells.

### Cell Fractionation

To minimize proteolysis, all buffers were supplemented with 5 mM Na_2_S_2_O_5_, 1 mM phenylmethylsulfonyl fluoride, 0.5 mM benzamidine, 20 µg/ml leupeptin, 10 µg/ml pepstatin A, 25 µg/ml aprotinin and 1 mM dithiothreitol. All steps were performed at 4°C in the presence of 1 mM Na_3_VO_4_ to inhibit phosphatase activity. About 1×10^7^ cells were mechanically harvested with a sterile plastic disposable cell scraper and recovered by centrifugation at 3000×*g* for 15 min. Then, the pellet was washed twice in 30 ml of PBS; cytoplasmic and nuclear extracts were prepared as reported by Kim and Tucker [10]. Briefly, the cell pellet was suspended in 1 ml of sucrose buffer (0.32 M sucrose, 3 mM CaCl_2,_ 2 mM (CH_3_COO)_2_Mg, 0.1 mM EDTA, 100 mM Tris-HCl, pH 8.0 and 0.5% (v/v) NP-40) and centrifuged at 900×*g* for 2 min. The pellet contained the nuclei; the supernatant was centrifuged again at 17,000×*g* for 15 min and the resulting supernatant was the cytoplasmic fraction. The nuclear pellet was washed with 1 ml of sucrose buffer lacking NP-40, centrifuged at 200×*g* for 2 min, suspended in 200–400 µl of 10% SDS, 3% DTT and sonicated for 30 sec. The NM was isolated according to Barboro *et al.* [11]. Western blot (WB) analyses demonstrated effective fractionation of the cells (Figure S1). Cytoplasmic, nuclear and NM fractions were then processed for one- or two-dimensional polyacrylamide gel electrophoresis (1D or 2D-PAGE), as reported below.

Protein concentrations were determined using the Bio-Rad (München, Germany) protein microassay with bovine serum albumin as a standard.

For confocal microscopy, the NM was extracted *in situ* as described by Zeng *et al*. [13]. Cells grown on coverslips were washed in PBS and extracted twice in CSK buffer (0.10 M NaCl, 0.30 M sucrose, 10 mM PIPES, pH 6.8, 3 mM MgC1_2_, 0.5% (v/v) Triton X-100) for 15 min each. The chromatin fraction was removed by digestion in CSK buffer with 50 mM NaCl (digestion buffer) containing 100 µg/ml DNase I (Roche, Mannheim, Germany) for 30 min, followed by extraction in digestion buffer containing 0.25 M (NH_4_)_2_SO_4_ for 10 min, then, the coverslips were fixed in 4% formaldehyde in PBS.

### Gel Electrophoresis and WB Analyses

All samples were delipidated, cleaned and solubilized for 1D or 2D-PAGE as previously described [14]. For 1D-PAGE, eight µg of proteins extracted from different cell fractions were loaded onto 8–14% linear gradient polyacrylamide gels and separated at 5 mA/gel for 16 h at a constant temperature of 12°C. Conventional high-resolution 2D-PAGE was performed as previously described [14] using in the second dimension an 8–14% linear gradient. 2D-phosphate-affinity-PAGE was performed on a second dimension 7.5% polyacrylamide gel, containing 25 µM polyacrylamide-bound Mn^2+^-Phos-tag™ ligand (Phos-tag AAL-107; Phos-tag Consortium, Hiroshima, Japan), according to Kimura *et al* [15]. The hnRNP K phosphorylation pattern was also analyzed by a multiplexed proteomic technology [16]. Briefly, gels were stained with Pro-Q Diamond (Molecular Probe Inc., Eugene, OR, USA), a fluorescent dye capable of sensitive detection of phosphorylated amino acid residues in proteins separated by 2D-PAGE. Following image acquisition, gels were stained with SYPRO Ruby (Molecular Probe Inc.) to visualize total proteins.

The proteins separated by 1D or 2D-PAGE were transferred to a Hybond-P membrane (GE Healthcare, Piscataway, NJ), and immunodetection was performed using mouse monoclonal antibodies anti-hnRNP K/J (33 ng/ml, sc-28380, Santa Cruz Biotechnology Inc., Santa Cruz, CA, USA), anti-AR (2 µg/ml, AR441, DAKO, Carpinteria, CA), anti-PSA (1 µg/ml sc-69664 Santa Cruz), anti-α-Tubulin (400 ng/ml, T 6074, SIGMA), anti-PARP-1/2 (500 ng/ml, sc-7150, Santa Cruz) and anti-lamin B (200 ng/ml, sc-6216, Santa Cruz). All the concentration quoted are value in the final reaction mixture. In the compartmentalization experiments, it was not possible to use an internal control to ensure equal gel loading because the major components of the NM undergo appreciable changes, as we have previously demonstrated [6]; therefore, the relative amounts of hnRNP K and AR were determined following a previously described quantitative method [6], [17]. The comparison of the relative amounts of the proteins was performed using Student’s *t*-test within the OriginPro 7.5 software, exporting the single values of each film.

Detection and alignment of spots present in all 2D-WBs were carried out digitizing the autoradiography films with a GS-800 densitometer (BioRad) under the same scanning conditions; then, the images were matched using the software package PDQuest (ver. 8.6, BioRad). The relative amount of each spot was obtained by normalizing the spot volume to the total density of all spots.

### Semi-quantitative Real-time PCR

Total RNAs were obtained from treated or non-treated LNCaP cells grown with 0.1 nM DHT and incubated with anti-androgens (10^−5^ M BIC or 10^−6^ M CPA) for 24 h. RNA was reverse transcribed with oligo(dT) primers and PSA mRNA expression was analyzed by semi-quantitative real-time PCR by using the following primer: sense 5′-ACGGATGCTGTGAAGGTCAT and antisense 5′-CTTTGGGGTCAAGAACTCCT. The relative expression of PSA gene was assessed in comparison with the housekeeping gene glyceraldehyde-3-phosphate dehydrogenase (GAPDH) amplified with the following primers: sense 5′-GAAGGTGAAGGTCGGAGT and antisense 5′-CATGGGTGGAATCATATTGGAA. cDNAs were amplified for 50 cycles using iQ Supermix (Bio-Rad, Richmond, CA) containing the intercalating agent SYBR Green in a two-step amplification scheme (95°C, 15 seconds and 60°C, 30 seconds). Fluorescence was measured during the annealing step on a Mastercycler Eppendorf realplex. Blank controls, that did not contain cDNA, were run in parallel. Each treatment was repeated two times and all samples were run in triplicate. Following amplification, melting curves with 80 steps of 15 seconds and a 0.5°C temperature increase per step were done to control for amplicon identity. Relative expression values with SEs and statistical comparison (unpaired two tailed *t*-test) were obtained using Qgene software [18].

### Confocal Laser Scanning Microscopy and Image Analysis

Analysis of the spatial relationship between AR and hnRNP K in the NM was performed on both treated and untreated LNCaP cells using triple–color confocal microscopy. The following antibodies were used: rabbit anti-hnRNP K (tissue culture supernatant diluted 1∶25, ab 52600, Abcam, Cambridge, UK), mouse anti-AR at a final concentration of 33 mg/ml (AR441, DAKO,), CF633-conjugated donkey anti-rabbit IgG (Biotium, Hayward, CA) and CF555-conjugated donkey anti-mouse IgG (Biotium), both at a final concentration of 40 µg/ml. Goat anti-lamin B at the concentration of 8 µg/ml (sc-6216 Santa Cruz) and Alexa 488-conjugated donkey anti-goat at the concentration of 40 µg/ml (Invitrogen, Carlsbad, CA) were employed to detect effective NM purification. All confocal images were collected according to the Nyquist criterion as 3D data sets (z-stacks) with a step size of 250 nm using an Olympus FV-500 laser scanning confocal microscope equipped with a 60×/1.4 NA oil-immersion objective. Image processing and co-localization analyses were performed as previously described [6] using WCIF ImageJ v1.43 software (http://www.uhnresearch.ca/facilities/wcif/imagej/) that provides both the Pearson’s correlation coefficient (R) and the co-occurrence Manders’ coefficients (M1 and M2). Three-dimensional measurements of proteins cluster sizes were carried out on image stacks as already reported [6].

## Results

### Effects of Anti-androgen Treatment on LNCaP Cell Growth

**Figure 1:**
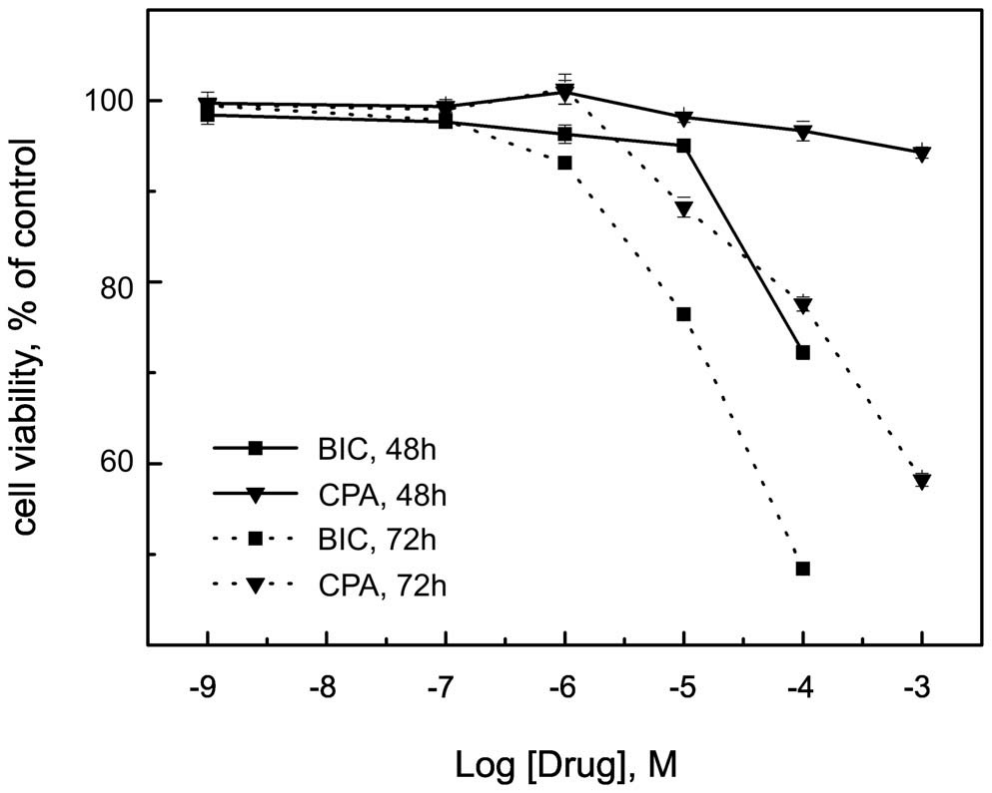
LNCaP cell proliferation in the presence of increasing concentrations of BIC or CPA. LNCaP cells were grown with 0.1-androgen under study. The effects were significant (P<0.01) for all concentrations ≥10^−6 ^M and for all times reported, except for 10^−5 ^M CPA for 48 h. Data are presented as percentage of the absorbance of BIC or CPA treated cells with respect to controls. The bars represent the mean ± SD of three independent experiments, each performed 10 times.

The data reported in Figure 1 show that in the presence of 0.1 nM DHT, both BIC and CPA inhibited LNCaP cell growth in a dose- and time-dependent fashion. After 72 h of treatment, the EC_50_, the concentration required for a 50% effect, were 1.53×10^−4^ and 26.38×10^−4^ for BIC and CPA, respectively. These results are consistent with previous investigations demonstrating that BIC and CPA cause a decrease in LNCaP cell viability in androgen-containing medium [19] and indicate that under these conditions, CPA is less potent than BIC. At 48 and 72 h, 10^−6 ^M CPA showed a very small, but significant (P<0.01), agonist effect. After treatment for 72 h with 10^−5 ^M BIC or 10^−4 ^M CPA, approximately 75% of cells were viable, indicating that at these concentrations, the two drugs had the same antagonistic effect. Thus, subsequent experiments were performed on cells treated for 72 h with 10^−5 ^M BIC or 10^−4 ^M CPA. The agonistic effects of 10^−6 ^M CPA were also studied.

### Cellular Localization and Modulation of AR and hnRNP K in BIC- or CPA-treated Cells

**Figure 2:**
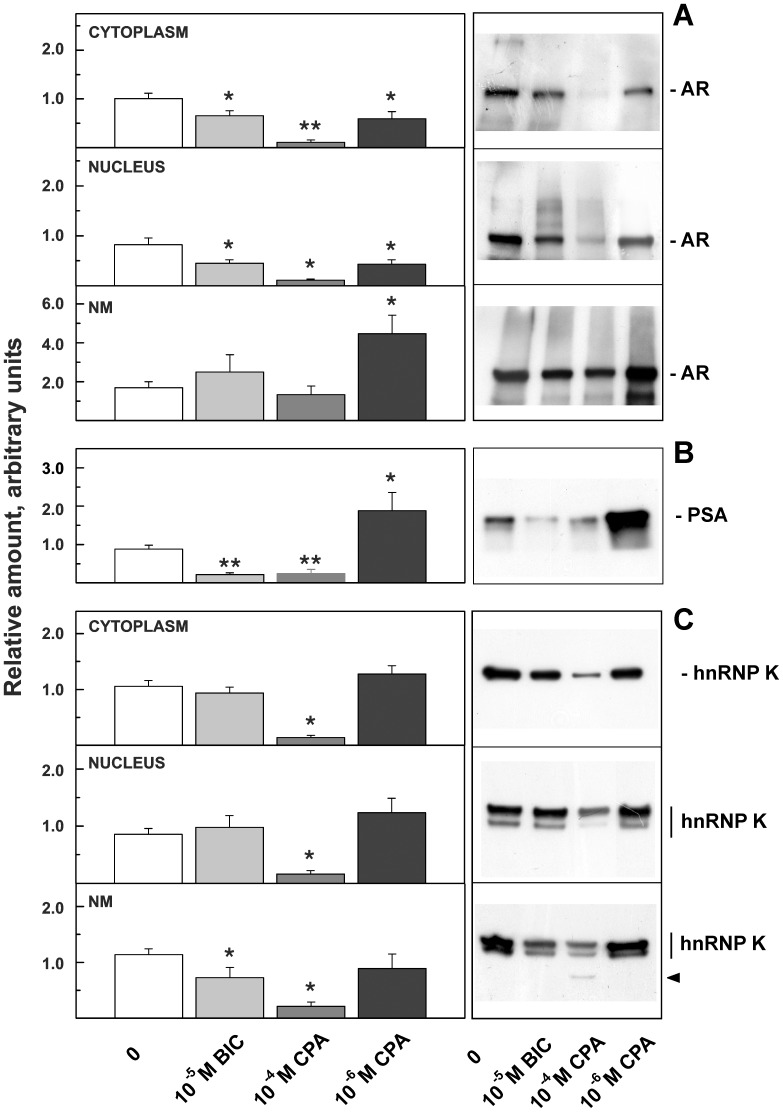
AR, PSA and hnRNP K expression in different cellular compartments after BIC or CPA treatment. 1D-WB analysis of samples isolated from LNCaP cells grown in the presence of 0.1 nM DHT, untreated (0) or treated for 72 h with 10^−5 ^M BIC, 10^−4 ^M and 10^−6 ^M CPA. Samples were probed with antibodies against AR (A), PSA (B) and hnRNP K (C). Each treatment was repeated at least three times. The ordinates represent the mean ± SE of the relative amounts of the proteins determined by quantitative analysis of three to seven WBs. The relative amounts were calculated as reported in the Materials and Methods section. Representative WBs are shown on the right. The arrowhead in (C) marks a proteolytic fragment of hnRNP K. *P<0.05, **P<0.01.

To study the effect of anti-androgen therapies on AR and hnRNP K, we analyzed the expression of these two proteins in three different subcellular compartments: the cytoplasm, the nucleus and the NM. The WB analyses reported in Figure 2A show that after BIC or CPA treatment, the AR was remarkably downregulated in both the cytoplasm and the nucleus. A concentration of 10^−5 ^M BIC or 10^−6^ M CPA caused comparable effects (approximately 50% reduction with respect to control), and at the higher concentration of CPA used the AR expression plummeted to 10% of control. In the previous section, we reported that LNCaP viability was equally affected by 10^−5^ M BIC and 10^−4 ^M CPA; therefore, these results indicate that in our experimental conditions, cellular growth was independent of AR expression. Interestingly, when the cells were supplemented with 10^−6^ M CPA, an approximately 2.5-fold increase was observed in the AR associated with the NM, and this increase was synchronous with higher intracellular PSA expression levels (Figure 2B). These results were confirmed examining the effect of the anti-androgens on the expression of the PSA mRNA gene by semi-quantitative real-time PCR. After 24 h of treatment a strong and significant down regulation of PSA mRNA was induced by 10^−5^ M BIC confirming the antagonistic behavior of the drug at this concentration (Figure S2). Vice versa, PSA gene expression was induced two-fold when the cells were supplemented with 10^−6^ M CPA in agreement with an agonistic effect (Figure S2).

These results suggest that a simple relationship between AR expression and the AR-responsive gene PSA does not exist, as we have already reported [6], and that NM-associated AR may represent the active complex. Hence, we examined hnRNP K compartmentalization. After BIC treatment, hnRNP K expression was significantly lower only within the NM (from 1.14 to 0.73; P = 0.05). When the cells were supplemented with 10^−4 ^M CPA, a strong decrease in hnRNP K expression was observed in all cellular compartments, and fragments of lower molecular weight were evident in the NM fraction, indicating extensive protein degradation in this nuclear compartment. At 10^−6^ M, CPA did not produce significant effects.

### Effect of BIC or CPA Treatment on NM Colocalization of AR and hnRNP K

**Figure 3:**
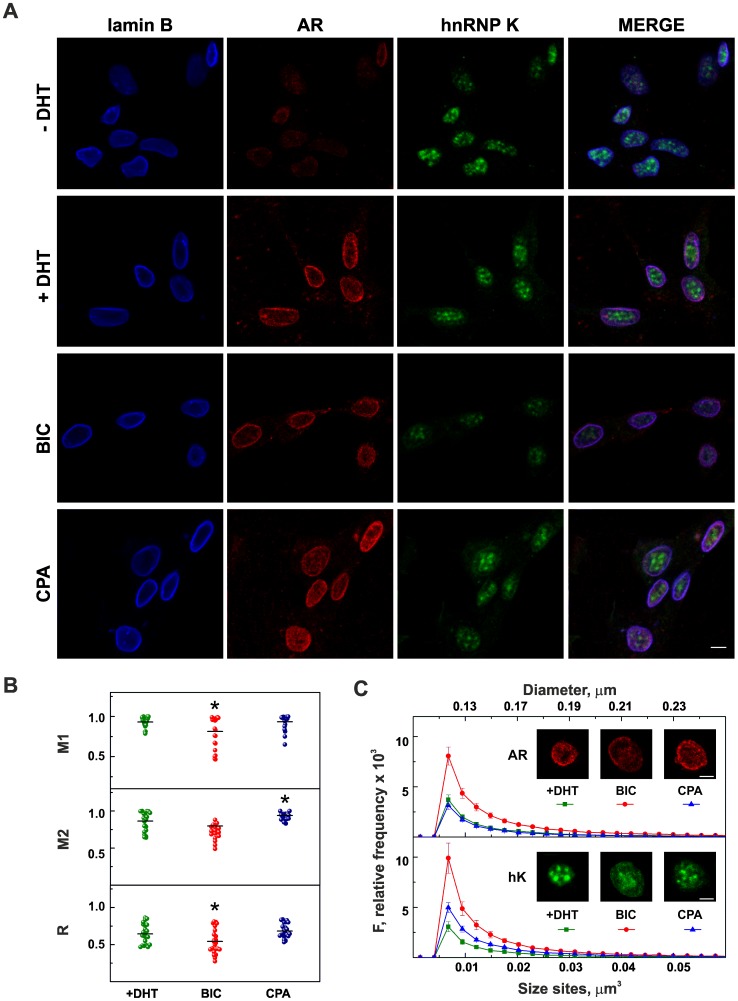
AR and hnRNP K are differently distributed in the NM after anti-androgen treatments. (A) Confocal microscopy analysis clearly shows that in the absence (−) of DHT, the AR is not localized in the NM, whereas in the presence (+) of 0.1 nM DHT, the AR is distributed both in the periphery and in inner punctuate sites. HnRNP K staining is independent of DHT and is present in the internal NM. After exposure of cells grown in presence of 0.1 nM DHT to 10^−5 ^M BIC, both AR and hnRNP K show a very weak diffuse fluorescence pattern. Treatment with 10^−6 ^M CPA gives rise to a strong increase in AR fluorescence. NMs were immunostained with anti-lamin B (blue), anti-AR (red) and anti–hnRNP K (green) antibodies. (B) Scatterplots show the quantification analysis of AR/hnRNP K co-localization. M1 corresponds to the fraction of AR overlapping hnRNP K, and M2 indicates the fraction of hnRNP K overlapping AR; R is Pearson’s coefficient. The horizontal lines indicate the mean values from 20–23 fields (160–190 total cells) replicated in two different experiments; *P<0.02. (C) Frequency distribution of the size of the punctuate sites, corresponding to AR (upper panel) and hnRNP K (lower panel), grouped in intervals of 0.003 µm^3^. The ordinates are the mean ± SE. The red curves representing BIC treatment are significantly different from control (+DHT; green curves P = 0.01). The blue curves correspond to CPA treatment. Representative projections of image stacks utilized to calculate the size of punctuate sites are reported. hK, hnRNP K. The bars correspond to 10 µm in (A) and 5 µm in (C).

Because the principal effects on AR and hnRNP K appear to implicate the NM, we performed confocal microscopy analysis in this subnuclear compartment to define the changes in AR/hnRNP K co-localization after anti-androgen treatment, utilizing BIC and CPA concentrations that did not induce NM protein degradation. Representative optical sections are shown in Figure 3A. A clean separation of the NM fraction was confirmed using an anti-lamin B antibody that, as expected, intensely stained a continuous rim of the peripheral NM, with reduced staining in the internal NM [11], [17], [20]. In LNCaP cells, grown in the absence of DHT, the AR was not localized in the NM, and only minimal fluorescence intensity was visible. In the presence of androgen, AR was distributed almost at the periphery, and some punctuate sites were observable in the internal NM, in agreement with previous investigations that showed a defined hyperspeckled subnuclear distribution in the high salt extraction-resistant fraction [21], [22]. HnRNP K was primarily localized in the internal NM in discrete spherical particles. Analysis of the immunofluorescence signals, performed as reported in the Materials and Methods section, confirmed that, in the presence of DHT, the co-localization between AR and hnRNP K, already demonstrated in the whole nucleus [6], persists in the NM (Figure 3B). The coefficients M1 (the fraction of AR overlapping hnRNP K) and M2 (the fraction of hnRNP K overlapping AR) were 0.93±0.01 and 0.86±0.03, respectively, demonstrating an excellent co-localization between the two proteins. Moreover, we obtained a Pearson’s coefficient (R) of 0.64±0.03, indicating a discrete correlation between their intensity distributions. After BIC treatment, whereas WB analysis indicated no changes in the level of AR associated with the NM, confocal microscopy showed a less intense and more uniform staining in the internal NM. In addition, hnRNP K fluorescence intensity was reduced, with a consequent decrease in M1 and R. When the cells were supplemented with 10^−6^ M CPA, in agreement with WB results, the AR intensity fluorescence was strongly increased. Consequently, an increase of the coefficient M2 (from 0.86±0.03 to 0.94±0.01, P<0.02) was observed, indicating a higher hnRNP K fraction overlapping the AR. Moreover, the AR was distributed in hyperspeckled sites, as after DHT treatment.

The number and size of hyperspeckled sites, determined on image stacks, confirmed these findings. The size frequency distribution curves reported in Figure 3C showed a maximum of 0.0075 µm^3^, corresponding to a sphere of 0.1 µm in diameter, a value very close to the dimension of nuclear speckles [23]. In addition, some larger sites were also present. After BIC treatment, the frequency of the smaller sites for both AR and hnRNP K approximately doubled as a consequence of major fragmentation of hyperspeckled sites, whereas the curves were superimposable after DHT and CPA treatments.

### Evaluation of hnRNP K Phosphorylation

**Figure 4:**
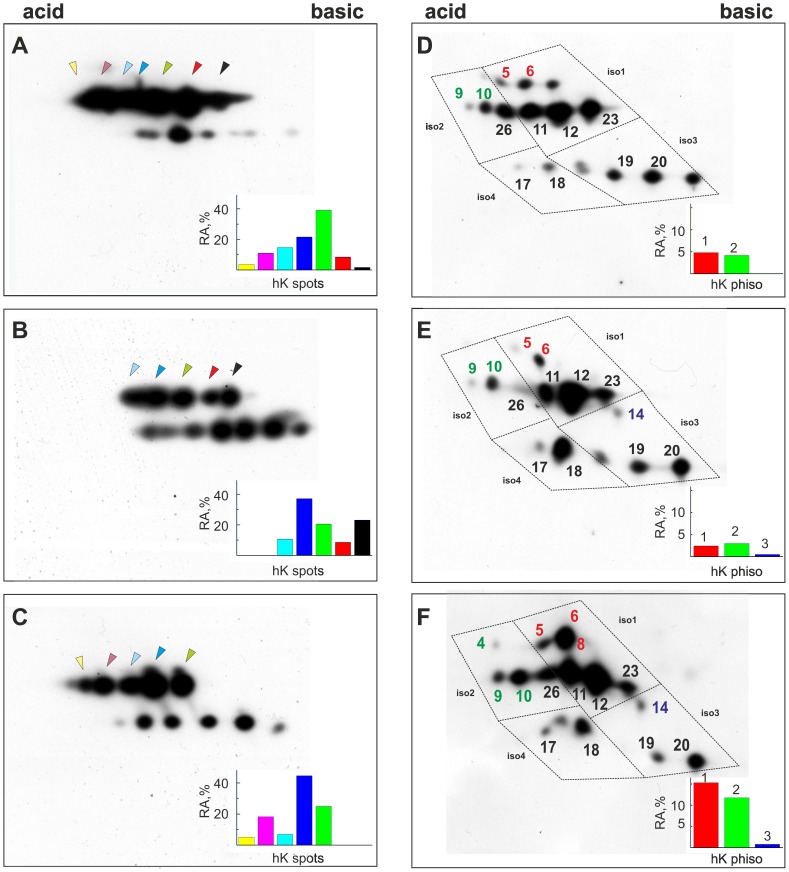
The phosphorylation status of hnRNP K changes after anti–androgen exposure. NMs extracted from LNCaP untreated cells grown in presence of 0.1(A, D), cells treated with 10^−5 ^M BIC (B, E) and cells treated with 10^−6 ^M CPA (C, F). The samples were separated by conventional 2D-PAGE (A, B, C) or 2D-phosphate-affinity-PAGE (D, E, F), as reported in the Materials and Methods section, and probed with the anti-hnRNP K antibody. Magnified sections of the hnRNP K detection region are shown. The arrowheads indicate the various protein isoforms. The colors in the histograms correspond to the isoforms present in cells after the different treatments. In (D, E, F), dotted squares surround the spots belonging to the four spliced forms (iso1, iso2, iso3 and iso4); the numbers that identify the spots are the same used by Kimura *et al* [15]. The histograms represent the relative amount (RA) of the hnRNP K spots marked with arrowheads in (A, B, C) and of the phosphorylated spots present in spliced isoform 1, 2, 3 in (D, E, F). The colors in the histograms correspond to spots. hK, hnRNP K; hK phiso, hnRNP K phosphorylated isoforms.

Because it is known that hnRNP K possesses multiple sites that can be phosphorylated in response to extracellular signals [24] and that the phosphorylation of these sites regulates its cellular localization, the interaction with molecular partners (DNA, RNA or proteins) and the transcriptional effects of the protein [25]–[27], we characterized hnRNP K phosphorylation after exposure to anti-androgen treatments. NM proteins isolated from control and treated cells were separated by 2D-PAGE and WB analyses were performed. Several spots corresponding to alternatively spliced forms of hnRNP K were visible (Figure 4A). After treatment with anti-androgens, a change in the relative quantity of the single spots was observed. In the cells supplemented with 10^−5 ^M BIC, some acidic species decreased, while they increased in the cells treated with 10^−6 ^M CPA, suggesting that the two drugs induced an opposite effect on hnRNP K phosphorylation (Figure 4B and C). In the presence of 10^−4 ^M CPA, several spots with a lower molecular weight were observed, indicating protein fragmentation (data not shown), as already evident in 1D-WB. Therefore, we examined the effects of BIC and the lower CPA concentration on different hnRNP K isoforms using phosphate-affinity electrophoresis, a technique able to detect phosphoproteins based on a shift in their electrophoretic mobility. As expected, multiple hnRNP K forms were observed (Figure 4D–F). Because the coding hnRNP K sequence is completely conserved between human and mice, it was possible to superimpose our maps on those of J774.1 cells characterized by Kimura *et al*. [15]. In untreated cells, four alternative spliced forms (designated 1, 2, 3 and 4) of hnRNP K were distinguishable; isoforms 1 and 2 were more abundant and partly phosphorylated, as indicated by the slower migration of spots 5 and 6. After treatment with 10^−5 ^M BIC, spot 26 (belonging to isoform 2) disappeared, and spots 5 and 6 (isoform 1) decreased, demonstrating hnRNP K hypophosphorylation. A parallel increase in spot 18 (isoform 4) and the appearance of the spot 14 (isoform 3) were also evident. In the presence of 10^−6 ^M CPA, a strong increase in spots 5, 6 and 8 (isoform 1), 4, 9, 10 (isoform 2) and 14 (isoform 3) occurred, demonstrating that this condition is associated with a global hyperphosphorylation of the protein. The spots that after anti-androgen treatment undergo major changes are expected to be phosphorylated at serine 116 (spots 4, 5, 6), serine 284 (spots 8) and serine 353 (spots 9, 10) [15]. It is known that these amino acids are modulated by external stimulation and are responsible for hnRNP K subcellular localization [24], [25] and consequently its transcriptional effects.

**Figure 5:**
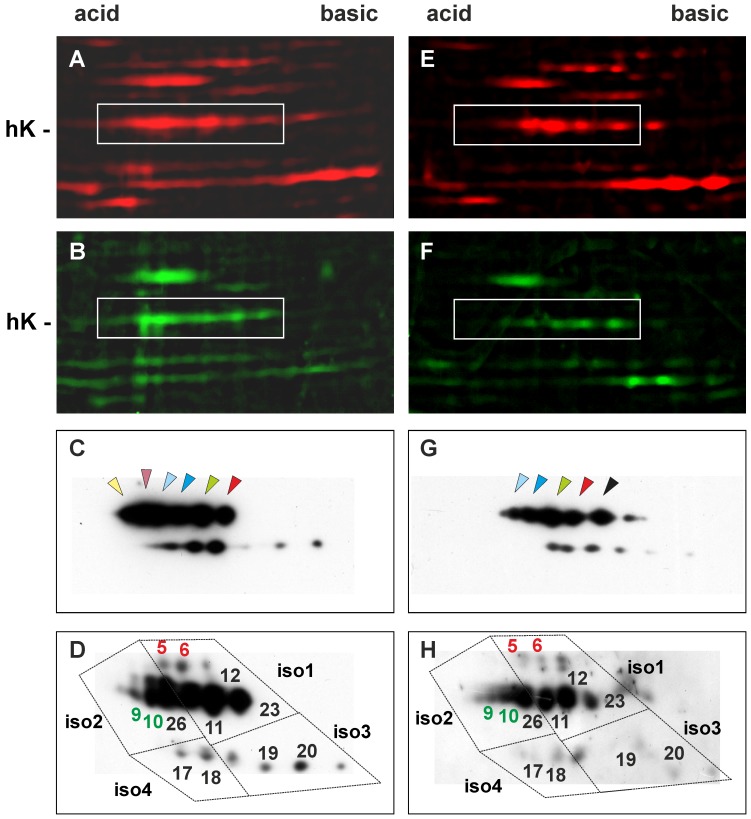
The PI3K/Akt inhibitor Wortmannin modifies hnRNP K phosphorylation status. LNCaP cells grown in presence of 0.1(A–D) control (E–H) Wortmannin treated cells. Magnified sections of 2D-PAGE stained with SYPRO Ruby (A, E) or Pro-Q Diamond, that selectively stains only phosphoproteins (B, F), are shown. The boxes mark the phosphorylated hnRNP K isoforms. After Wortmannin treatment a marked decrease in some hnRNP K isoforms and the loss of the more acidic spots are evident. The hnRNP K spots separated by 2D-PAGE (C, G) or 2D-phosphate-affinity-PAGE (D, H) and probed with anti-hnRNP K antibody are also reported. The arrowheads in C, G and the numbers in D, H indicate the different protein isoforms as in Figure 4. hK, hnRNP K.

As Akt kinase activity is elevated in LNCaP cells [28] and has been significantly correlated with PCa progression [29], to confirm that the changes observed in the 2D profiles depend on modifications of the hnRNP K phosphorylation status, we treated LNCaP cells with Wortmannin, an inhibitor of PI3K/Akt phosphorylation [30]. Under these conditions, a marked decrease in some hnRNP K isoforms and the loss of the more acidic spots occurred, indicating that these species correspond to phosphorylated protein (Figure 5 A, B, E and F). Moreover, as expected, 2D-phosphate-affinity-PAGE analysis showed an evident decrease of the spots with a lower electrophoretic mobility namely the spots 5, 6 (isoform 1) and 9, 10 (isoform 2) (Figure 5 D and H). These spots are those that underwent the major changes after BIC or CPA treatments (Figure E and F).

These results support the hypothesis that hnRNP K, and above all its phosphorylation, plays an important role in the response to anti-androgen treatments.

## Discussion

The current study shows that there is a strong relationship between the level of AR localized in the NM and both cell viability and PSA expression, indicating that AR transcriptional activity is critically dependent on its subnuclear compartmentalization. Moreover, the agonistic/antagonistic activity of anti-androgens is associated with modifications in the phosphorylation status of hnRNP K, a protein that directly interacts with the AR.

It is well recognized that LNCaP cell proliferation is especially stimulated by 0.1 nM DHT, and in the presence of anti-androgen treatment, cell proliferation decreases. Here, we report that, under the same conditions of viability, CPA is more potent than BIC in inhibiting AR transactivation function. This effect is associated with a strong degradation of hnRNP K. A peculiar characteristic of LNCaP cells is the presence of a point mutation in the ligand-binding domain in the AR gene at codon 877 (ACT-GCT), which results in the transition of a threonine into an alanine [31]. CPA shows a slightly increased affinity for the mutant receptor, whereas the binding affinity of BIC is not influenced by the mutation [31]. This information could explain the different AR stability as consequence of two different anti-androgen treatments; however, this difference does not influence cell proliferation, which is not easy to explain. In the presence of 10^−4 ^M CPA, the level of AR in the cytoplasm and the nucleus was approximately 4 times lower than that present after treatment with 10^−5 ^M BIC, whereas there was no significant difference in the AR associated with the NM. Therefore, this AR fraction could be sufficient to determine LNCaP proliferation. These results are in agreement with the notion that NM, in particular the internal NM, contains acceptor sites for AR [32], and binding to these sites is necessary for the biological activity of the receptor [33]. Therefore, the association/dissociation with the NM rather than AR degradation could be critical for hormone action. In agreement with this, Gonor *et al.* [34] have demonstrated that androgen responsiveness in PCa does not correlate with the presence or the levels of AR in cancer tissues but with the concentration of NM-bound AR.

In addition, the agonist activity displayed by CPA appears to be strongly bound to the amount of AR associated with the NM; in fact, an elevated PSA expression, found by WB analysis and real time PCR, is synchronous with a significant increase in the proportion of AR in the NM as detected by WB analysis and confocal microscopy. Hyperspeckled sites in the internal NM are visible after treatment with 10^−6 ^M CPA, as opposed to BIC exposure, which showed a diffuse and weak fluorescence. These results suggest that the hyperspeckled distribution of AR may be considered a consequence of interactions with the NM [22]. Hyperspeckled AR could correspond to nuclear speckles, the interchromatin granule clusters involved in RNA metabolism and processing. Nuclear speckles have relatively fixed positions and interact with the filament network of the internal NM; however, the proteins within them are very dynamic and can move quickly throughout the nuclear space [35]. Many active genes are located at the periphery of nuclear speckles, and the interaction between chromatin and the proteins present in these megacomplexes occurs via specific DNA sequences called matrix attachment regions (MARs) that tend to be concentrated in nuclear speckles. The MARs play an important role in several nuclear processes including organizing chromatin loops, augmenting gene expression and facilitating replication [36]. The interaction between NM proteins and MAR sequences is a dynamic event dependent on cellular differentiation: i.e., in androgen-independent PC3 cells, a smaller number of proteins bind the MARs compared with LNCaP cells [17]. Moreover, Buttyan *et al*. [37] have demonstrated not only that sex steroid complexes recognize specific acceptor sites on the NM of their respective target tissues but also that the DNA associated with the NM is at least in part responsible for the binding specificity. Therefore, the hyperspeckled distribution of AR could correspond to its transcriptionally active form into the NM that could directly, or by the interaction with other proteins, bind MAR sequences.

Heterogeneous nuclear ribonucleoproteins (hnRNP), including hnRNP K, are among the most abundant components of the NM and share in the stabilization of the internal NM network [11], [35], [38]. Moreover, hnRNP K is one among the NM proteins that bind DNA-MARs [17], [39] and has been localized in the interchromatin granule clusters [40]. AR stabilization in hyperspeckled structures could, at least in part, depend on its interaction with hnRNP K.

We have shown that a phosphorylated isoform of hnRNP K is overexpressed in the NM extracted from prostate carcinoma tissues [7], [41]. hnRNP K expression was associated in a significant way with the degree of tumor differentiation (Gleason score) [42], with an increased risk of PSA progression and finally, with a higher probability of death [43]. Here, we demonstrated that CPA or BIC can induce an opposite effect on phosphorylation status of the hnRNP K localized in the NM. An agonistic effect caused an increase, and an antagonist effect caused a decrease in the phosphorylated hnRNP K isoforms. Synchronously, a change in the co-localization between AR/hnRNP K as well in the dimensions of speckles was observed. Because hnRNP K phosphorylation regulates its interaction with DNA, RNA and protein patterns [24], [26], the hyperphosphorylation that we have demonstrated to occur when CPA exerts an agonist effect could support the role of hnRNP K AR transcriptional co-activator similar to when phosphorylated hnRNP K acts as a cofactor for p53-mediated transcription [44].

In conclusion, our data provide evidence that the transcriptionally active form of AR is localized into the NM, and the bond between AR and hnRNP K depends on the phosphorylation of the latter. The bond between hnRNP K and AR facilities the stabilization of the latter into the NM and/or its interaction with MAR sequences and promoted transcriptional activity. Treatments with agonist/antagonist drugs, modifying the level of hnRNP K phosphorylation, increased/decreased its interaction with AR and consequently its activity.

Work is in progress in our laboratory, using specific inhibitors of the kinase activities, to define the potential therapeutic and/or chemopreventive role of hnRNP K hypophosphorylation in PCa progression and in androgen resistance.

## Supporting Information

Figure S1 **Cell fractionation effectively separates cellular compartments.** Eight µg of proteins extracted from whole cells (W), cytoplasm (Cyto), nucleus (Nu) and nuclear matrix (NM) were immunoblotted for α-Tubulin, PARP or lamin B as indicated. α-Tubulin was found only in the cytoplasmic fraction, the nuclear marker PARP was present in the nucleus and in small quantity in the NM as reported by Kaufmann et al [12] but was completely absent in the cytoplasm and finally lamin B was enriched in the NM fraction with respect to the nucleus and completely absent in the cytoplasm.
(TIF)

Figure S2 **Effects of BIC or CPA exposure on PSA mRNA expression.** LNCaP cells grown in presence of 0.1 nM DHT were treated for 24 h with 10^−5 ^M BIC or 10^−6 ^M CPA and real time semi-quantitative PCR carried out as reported in Materials and Methods. Mean normalized expression values were calculated by comparison with housekeeping gene GAPDH amplified in parallel. Two treatments were performed and all amplifications were done in triplicate. Error bars correspond to SE.
(TIF)
