# Supplementary figures and images for: Androgen Receptor Activity Is Affected by Both Nuclear Matrix Localization and the Phosphorylation Status of the Heterogeneous Nuclear Ribonucleoprotein K in Anti-Androgen-Treated LNCaP Cells
Source: PLoS One. 2013 Nov 13;8(11):e79212. doi: 10.1371/journal.pone.0079212 (PMC3827347; doi:10.1371/journal.pone.0079212)

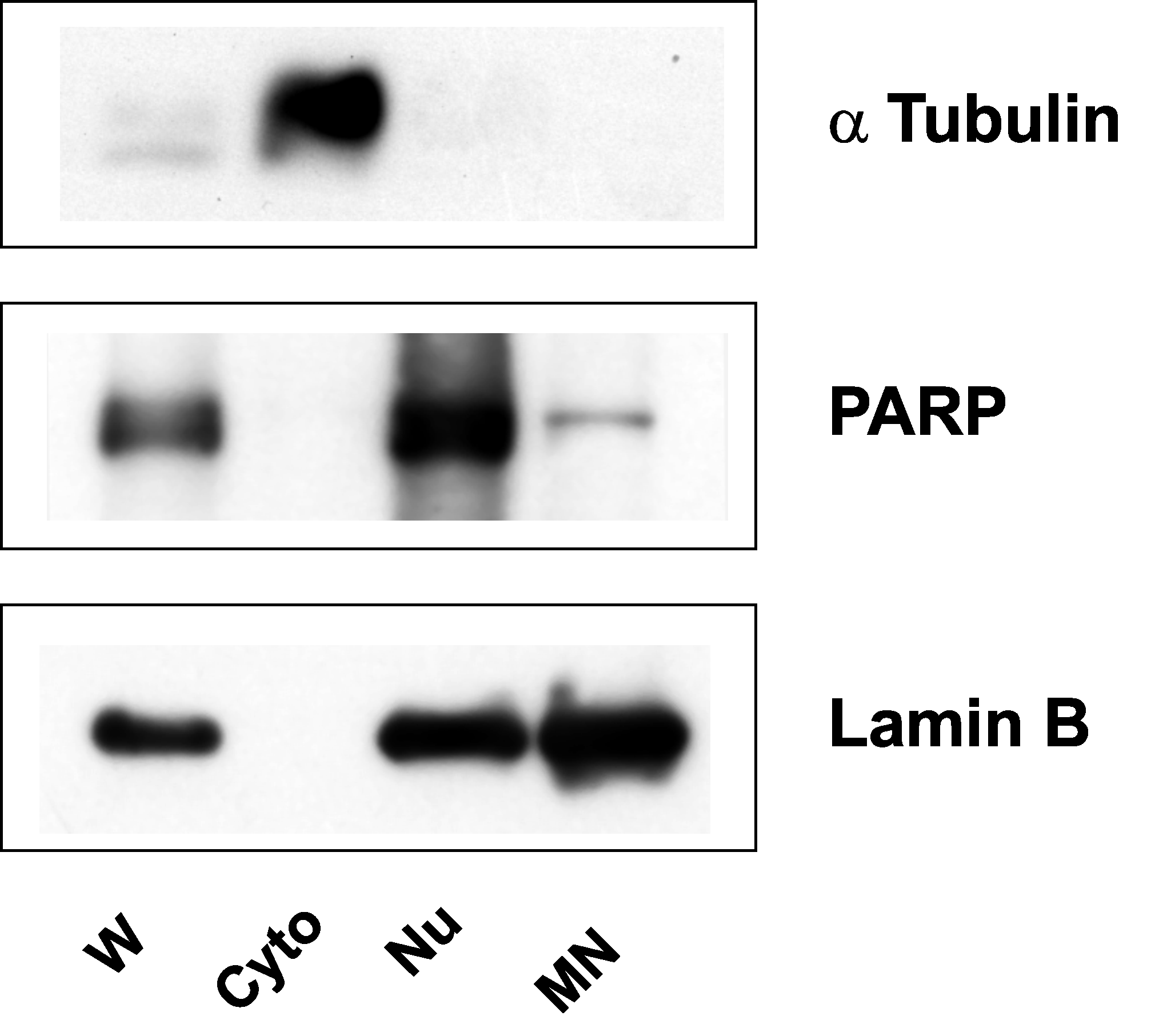

Supplement: Figure S1 — Cell fractionation effectively separates cellular compartments. Eight µg of proteins extracted from whole cells (W), cytoplasm (Cyto), nucleus (Nu) and nuclear matrix (NM) were immunoblotted for α-Tubulin, PARP or lamin B as indicated. α-Tubulin was found only in the cytoplasmic fraction, the nuclear marker PARP was present in the nucleus and in small quantity in the NM as reported by Kaufmann et al [12] but was completely absent in the cytoplasm and finally lamin B was enriched in the NM fraction with respect to the nucleus and completely absent in the cytoplasm. (TIF) [file pone.0079212.s001.tif]

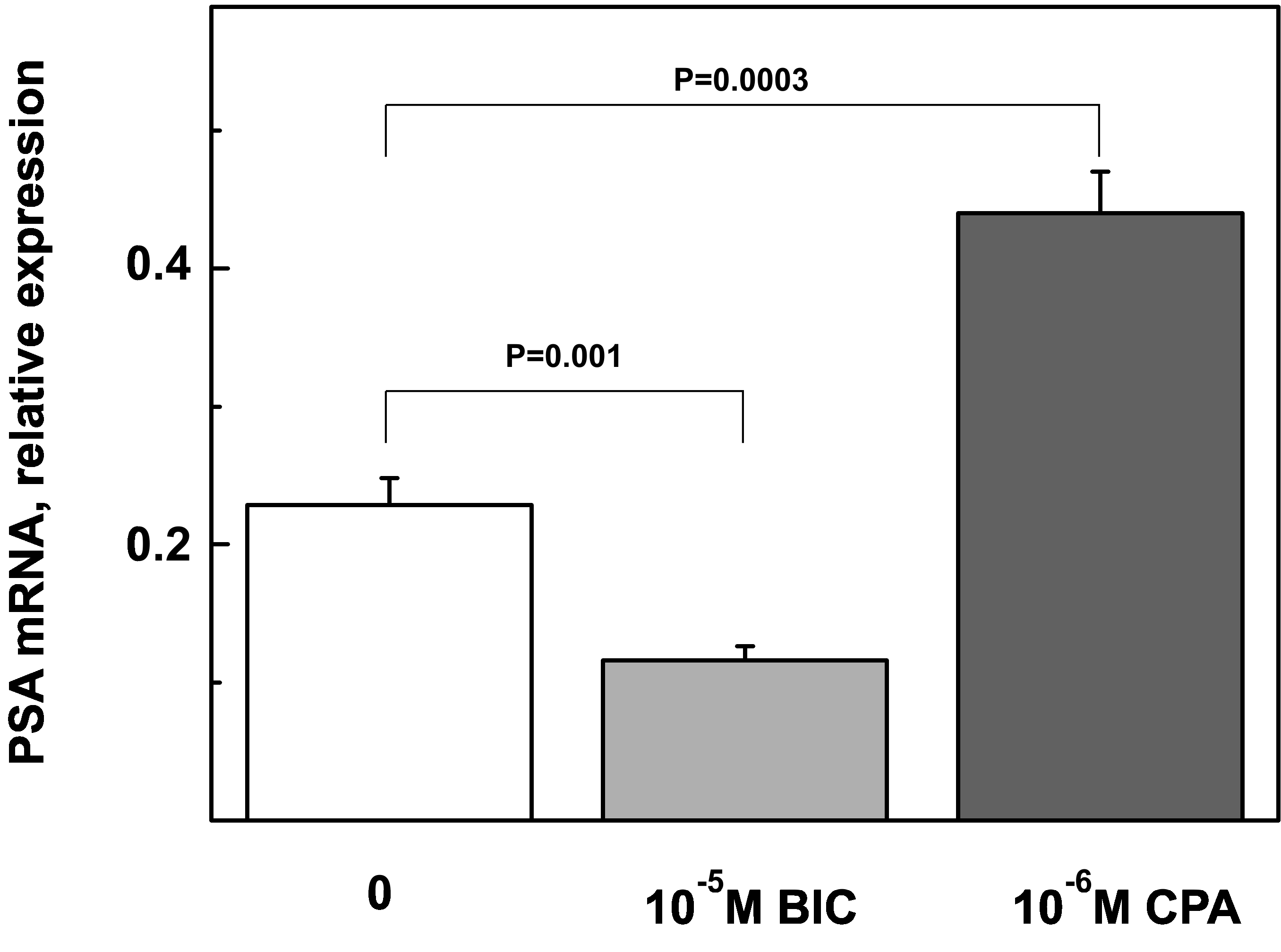

Supplement: Figure S2 — Effects of BIC or CPA exposure on PSA mRNA expression. LNCaP cells grown in presence of 0.1 nM DHT were treated for 24 h with 10−5 M BIC or 10−6 M CPA and real time semi-quantitative PCR carried out as reported in Materials and Methods. Mean normalized expression values were calculated by comparison with housekeeping gene GAPDH amplified in parallel. Two treatments were performed and all amplifications were done in triplicate. Error bars correspond to SE. (TIF) [file pone.0079212.s002.tif]
